# Predictors of Mortality in Patients with Hepatic Encephalopathy: A Retrospective Analysis

**DOI:** 10.7759/cureus.87677

**Published:** 2025-07-10

**Authors:** Zafeer-ul-Hassan Iqbal, Ayesha Pervaiz, Fatima Chaudhry, Zunaira Shakeel, Shahzaib Hassan, Himat Ali Faiaz, Shahnoor Aleem

**Affiliations:** 1 Internal Medicine, District Headquarter Hospital, Rawalpindi, PAK; 2 Internal Medicine, Punjab Rangers Teaching Hospital, Lahore, PAK; 3 Internal Medicine, Sundus Foundation, Lahore, PAK; 4 Internal Medicine, District Headquarter Hospital, Dera Ghazi Khan, PAK; 5 Internal Medicine, Federal Medical College, Islamabad, PAK; 6 Internal Medicine, Hazrat Bari Imam Sarkar (HBS) Medical and Dental College, Islamabad, PAK

**Keywords:** hepatic encephalopathy, liver, mortality, neuropsychiatric syndrome, serum ammonia

## Abstract

Background

Hepatic encephalopathy (HE) is a serious neuropsychiatric complication of liver dysfunction associated with significant morbidity and mortality.

Objective

The objective of this study was to evaluate the predictors of in-hospital mortality in patients with HE through retrospective analysis.

Methods

This retrospective observational cohort study was conducted at Punjab Rangers Teaching Hospital, Lahore, Pakistan, from January 2023 to January 2025. A total of 201 adult patients (≥18 years) admitted with a diagnosis of hepatic encephalopathy were included in the study. The diagnosis of HE was made clinically based on the West Haven criteria and supported by biochemical and radiological assessments as needed. Patients with both acute and chronic liver disease were considered, provided they fulfilled the diagnostic criteria for HE. Data were extracted from the hospital’s electronic medical record system using a structured questionnaire. Clinical and laboratory parameters were analyzed to identify predictors of in-hospital mortality using multivariate logistic regression.

Results

The in-hospital mortality rate was 57 (28.4%). Non-survivors had significantly higher Model for End-Stage Liver Disease (MELD) scores (mean 29.3 vs. 24.9, p < 0.001), elevated serum ammonia levels (mean 136.2 µmol/L vs. 103.1 µmol/L, p < 0.001), more frequent renal dysfunction (37 (64.9%) vs. 41 (28.9%), p < 0.001), and hyponatremia (33 (57.9%) vs. 43 (29.6%), p = 0.002). High-grade HE (Grade III/IV) was more prevalent among non-survivors (42 (73.7%) vs. 52 (36.2%), p < 0.001). Independent predictors of mortality included MELD score ≥28, serum ammonia ≥120 µmol/L, renal dysfunction, serum sodium <130 mEq/L, and high-grade HE. The MELD score demonstrated the highest discriminatory ability (area under the curve (AUC) = 0.78).

Conclusion

In patients with HE, higher MELD scores, elevated serum ammonia, renal impairment, hyponatremia, and advanced HE grades are significant predictors of mortality. These findings highlight the importance of early risk assessment and may guide decisions regarding intensive management and liver transplant referral.

## Introduction

Hepatic encephalopathy (HE) is a complex neuropsychiatric syndrome arising as a consequence of liver failure and portosystemic shunting, characterized by cognitive dysfunction, altered mental status, and, in severe cases, coma. Although it can potentially be reversed at the beginning, it has a poor prognosis if not treated or if it has repeated episodes [1]. The effect of any liver disease around the world is significantly enhanced through HE, and it is very common among those with decompensated cirrhosis or a failure of the liver [2]. As a major complication of liver dysfunction, HE frequently serves as a sign of irreversible liver deterioration and has high short- and long-term mortality rates for the patients [3]. The underlying mechanism of HE is due to the cumulative effect of underlying dysfunction of the liver, intensified production of ammonia from the intestines, systemic inflammation, and disruption of the blood-brain barrier. Among them, raised blood ammonia levels are especially important as an important cause [4]. However, hyperammonemia does not explain the full amplitude of neurological deficit observed in HE, suggesting other systemic and molecular mechanisms are critical, including cytokine release, endotoxemia, and oxidative stress. Grading is done in the West Haven Criteria (mild neuropsychiatric symptoms-I to deep unconsciousness-IV) [5].

The reason for the mortality in HE is not just the encephalopathy but the complications that follow. Diseases such as sepsis, renal failure, gastrointestinal bleeding, and aspiration pneumonia account for mortality amongst HE patients [6]. With this in mind, it is pivotal that one ought to know the indicators of adverse outcomes in order to have timely and effective intervention from medical personnel. Clinicians routinely employ standardized scoring systems (such as the Child-Turcotte-Pugh (CTP) score and the Model for End-Stage Liver Disease (MELD)) to measure the severity of liver disease and order of priority for liver transplantation [7]. These tools can provide an idea of how patients should be doing, but they are not accurate in calculating mortality in HE cases. There is increasing evidence that some markers can serve to predict the higher mortality in patients who experience HE [8]. Major predictors include high serum ammonia levels, high MELD or MELD-Na values, indications of renal impairment with high creatinine, hyponatremia, systemic inflammatory markers, including C-reactive protein (CRP)q or white blood cell count, and Acute-On-Chronic Liver Failure (ACLF) scores [9]. Besides, it has been proved that the presence of cerebral edema in MRI and CT, or electroencephalographic changes, also has additional value in predicting the outcome [10]. As a matter of fact, the outcomes and clinical course of HE vary depending on whether it is associated with acute liver failure (ALF), chronic liver disease (CLD), or ACLF [11]. In ALF, the onset of HE may be rapid with cerebral edema and increased intracranial pressure, requiring urgent intensive care and possible urgent liver transplantation. Recurrent episodes of HE occur frequently in people with CLD, including cirrhosis, and are closely linked to multi-organ failure, malnutrition, and sarcopenia [12]. Since ACLF is acquiring the status of a well-defined clinical entity, HE is one of the key diagnostic markers contributing significantly to the increased short-term mortality in these patients [13].

Objective

This study aimed to evaluate the predictors of in-hospital mortality in patients with HE through retrospective analysis. The primary outcome of the study was in-hospital mortality, defined as death occurring during the same hospital stay in which hepatic encephalopathy was diagnosed. Patients who were discharged after clinical recovery were considered survivors. The association of various clinical and laboratory parameters with mortality was examined to identify potential predictors.

## Materials and methods

Methodology

This retrospective observational cohort study was conducted at Punjab Rangers Teaching Hospital, Lahore, Pakistan, from January 2023 to January 2025. A total of 201 adult patients (≥18 years) admitted with a diagnosis of HE were included in the study. The diagnosis of HE was made clinically based on the West Haven criteria [5] and supported by biochemical and radiological assessments as needed. Patients with both acute and chronic liver disease were considered, provided they fulfilled the diagnostic criteria for HE.

Inclusion and exclusion criteria

Eligible participants must be at least 18 years of age and have a diagnosis of HE (Grades I-IV) established either at the time of hospital admission or during their subsequent hospitalization. Furthermore, comprehensive medical records encompassing laboratory investigations, imaging studies, and outcomes data must be available for review. A documented etiology of the underlying liver disease (such as viral hepatitis, alcohol-related liver disease, or non-alcoholic steatohepatitis) is also required.

Conversely, patients will be excluded if their altered mental status is attributed to non-hepatic causes, including drug intoxication, primary neurological disorders, or other metabolic encephalopathies. Individuals who have undergone liver transplantation are also excluded. Additional exclusions apply to patients whose clinical data are deemed insufficient or incomplete for analysis (patients who lacked essential laboratory values such as serum ammonia, creatinine, or MELD score components or whose imaging and discharge summary were missing), as well as those who were discharged against medical advice prior to achieving clinical stabilization or before a definitive clinical outcome could be determined.

Data collection

Data were extracted from the hospital’s electronic medical record system using a structured questionnaire. Data included patient demographics (age, sex, comorbidities), liver disease history (etiology, previous HE episodes, variceal bleeding, ascites), and clinical presentation (grade of HE, Glasgow Coma Scale, presence of precipitating factors such as infection, gastrointestinal bleeding, or electrolyte imbalance). Laboratory values, including serum ammonia, bilirubin, creatinine, sodium, albumin, international normalized ratio (INR), CRP, and complete blood count, were recorded. Severity scores such as MELD, MELD-Na, CTP, and ACLF grades (where applicable) were calculated. Radiological assessments were reviewed to identify cerebral edema or portosystemic shunts when imaging was available.

Statistical analysis

Data were analyzed using IBM SPSS Statistics software, version 26 (IBM Corp., Armonk, NY). Continuous variables were summarized using means and standard deviations (SD) or medians with interquartile ranges (IQR), depending on the distribution. Categorical variables were presented as counts and percentages. Variables found to be significantly associated with mortality (p < 0.1) were included in a multivariate logistic regression model to identify independent predictors of mortality. A p-value less than 0.05 at a 95% confidence interval (CI) was considered statistically significant.

## Results

Data were collected from 201 patients with a mean age of 56.8 ± 11.3 years, with 137 (68.2%) being male. The most common underlying etiology was alcohol-related cirrhosis in 83 (41.3%) patients, followed by hepatitis C in 45 (22.4%), hepatitis B in 28 (13.9%), and non-alcoholic fatty liver disease in 23 (11.4%). Previous episodes of hepatic encephalopathy were reported in 62 (30.8%) patients. Severe HE (Grade III or IV) was present in 93 (46.3%) cases. Infections were the leading precipitating factor, observed in 97 (48.2%) patients, followed by gastrointestinal bleeding in 46 (22.9%), electrolyte imbalance in 39 (19.4%), and constipation in 33 (16.4%). The most common disturbances included hyponatremia (serum sodium <130 mEq/L) in 33 (84.6%) patients, hypokalemia (serum potassium <3.5 mEq/L) in 21 (53.8%), and hypocalcemia (corrected serum calcium <8.5 mg/dL) in nine (23.1%) patients. Some patients had multiple concurrent imbalances. The mean MELD score was 26.2 ± 6.8, and the mean serum ammonia level was 114.7 ± 42.5 µmol/L. Renal dysfunction was defined as the presence of serum creatinine ≥1.5 mg/dL, an acute increase in creatinine of ≥0.3 mg/dL from baseline in patients with known chronic kidney disease (CKD), or the need for dialysis during hospitalization. Based on these criteria, renal dysfunction was observed in 79 patients (39.3%). The mean serum sodium level among the study population was 130.5 ± 5.9 mEq/L (Table 1).

**Table 1:** Baseline characteristics NAFLD: non-alcoholic fatty liver disease; HE: hepatic encephalopathy; MELD: Model for End-Stage Liver Disease, GI: gastrointestinal Values are expressed as mean ± standard deviation (SD) or frequency (n) with percentage (%), as appropriate.

Variable	Value
Number of patients	201
Mean age (years)	56.8 ± 11.3
Male	137 (68.2%)
Alcohol-related cirrhosis	83 (41.3%)
Hepatitis C	45 (22.4%)
Hepatitis B	28 (13.9%)
NAFLD	23 (11.4%)
Previous HE episodes	62 (30.8%)
Grade III/IV HE	93 (46.3%)
Infection as precipitant	97 (48.2%)
GI bleeding	46 (22.9%)
Electrolyte imbalance	39 (19.4%)
Constipation	33 (16.4%)
Mean MELD score	26.2 ± 6.8
Serum ammonia (µmol/L)	114.7 ± 42.5
Renal dysfunction	79 (39.3%)
Serum sodium (mEq/L)	130.5 ± 5.9

A MELD score ≥28 was associated with an odds ratio (OR) of 2.9 (95% CI: 1.5-5.8, p = 0.002). Serum ammonia levels ≥120 µmol/L showed an OR of 2.3 (95% CI: 1.2-4.5, p = 0.01), while the presence of renal dysfunction increased the risk of mortality with an OR of 3.4 (95% CI: 1.7-6.9, p < 0.001). Patients presenting with Grade III/IV encephalopathy had an OR of 2.6 (95% CI: 1.3-5.1, p = 0.006), and those with serum sodium <130 mEq/L had an OR of 2.1 (95% CI: 1.1-4.0, p = 0.022), indicating a significantly higher risk of death (Table 2).

**Table 2:** Predictors of mortality MELD: Model for End-Stage Liver Disease; HE: hepatic encephalopathy Multivariate logistic regression was used. Odds ratios (ORs) with 95% confidence intervals (CIs) are reported. A p-value <0.05 was considered statistically significant.

Variable	Odds ratio (OR)	95% CI	p-value
MELD score ≥28	2.9	1.5–5.8	0.002
Serum ammonia ≥120 µmol/L	2.3	1.2–4.5	0.01
Renal dysfunction	3.4	1.7–6.9	<0.001
Grade III/IV HE	2.6	1.3–5.1	0.006
Serum sodium <130 mEq/L	2.1	1.1–4.0	0.022

When comparing survivors (n = 144) to non-survivors (n = 57), several significant differences were observed. The mean age was slightly higher among non-survivors (58.9 ± 12.4 years) compared to survivors (55.9 ± 10.8 years), though not statistically significant (p = 0.18). High-grade hepatic encephalopathy (Grade III/IV) was significantly more frequent in non-survivors (42 (73.7%)) than in survivors (52 (36.2%), p < 0.001). The mean MELD score was significantly higher in non-survivors (29.3 ± 6.2) compared to survivors (24.9 ± 6.4), and similarly, serum ammonia levels were elevated in non-survivors (136.2 ± 38.9 µmol/L) versus survivors (103.1 ± 39.8 µmol/L), both with p-values <0.001. Renal dysfunction was observed in 37 (64.9%) of non-survivors, compared to 41 (28.9%) of survivors (p < 0.001). Additionally, serum sodium levels below 130 mEq/L were more common among non-survivors (33 (57.9%)) than survivors (43 (29.6%), p = 0.002). Infection as a precipitating factor was significantly associated with mortality, present in 35 (61.4%) of non-survivors compared to 61 (42.4%) of survivors (p = 0.004) (Table 3).

**Table 3:** Comparison of survivors vs. non-survivors HE: hepatic encephalopathy; MELD: Model for End-Stage Liver Disease Values are expressed as mean ± standard deviation (SD) or frequency (n) with percentage (%), as appropriate. Independent t-test was used for continuous variables; chi-square test was used for categorical variables. A p-value <0.05 was considered statistically significant.

Variable	Survivors (n=144)	Non-Survivors (n=57)	p-value
Mean age (years)	55.9 ± 10.8	58.9 ± 12.4	0.18
Male	95 (66.0%)	42 (73.7%)	0.26
Grade III/IV HE	52 (36.2%)	42 (73.7%)	<0.001
Mean MELD score	24.9 ± 6.4	29.3 ± 6.2	<0.001
Serum ammonia (µmol/L)	103.1 ± 39.8	136.2 ± 38.9	<0.001
Renal dysfunction	41 (28.9%)	37 (64.9%)	<0.001
Serum sodium <130 mEq/L	43 (29.6%)	33 (57.9%)	0.002
Infection as precipitant	61 (42.4%)	35 (61.4%)	0.004

Grade I was observed in 42 (20.9%) patients with a mortality rate of one (2.3%), while Grade II occurred in 66 (32.8%) patients with a mortality rate of nine (13.6%). Grades III and IV were seen in 57 (28.4%) and 36 (17.9%) patients, respectively, with mortality rates of 20 (35.1%) and 24 (66.7%), indicating a strong correlation between HE grades and outcome (Table 4).

**Table 4:** Distribution of HE grades and mortality HE: hepatic encephalopathy

HE grade	Number of patients (n, %)	Mortality (n, %)
Grade I	42 (20.9%)	1 (2.3%)
Grade II	66 (32.8%)	9 (13.6%)
Grade III	57 (28.4%)	20 (35.1%)
Grade IV	36 (17.9%)	24 (66.7%)

Regarding precipitating factors, infection was the most common, seen in 97 (48.2%) patients, and had the highest associated mortality at 37 (38.1%). Gastrointestinal bleeding and electrolyte imbalance were noted in 46 (22.9%) and 39 (19.4%) patients, with mortalities of 15 (32.6%) and 11 (28.2%), respectively. Constipation (33 (16.4%)), excess protein intake (21 (10.4%)), and sedative use (18 (9.0%)) were less common, with mortality rates ranging from three (14.3%) to five (27.8%). In 27 patients (13.4%), no clear precipitant was identified, and the mortality in this subgroup was seven (25.9%) (Table 5).

**Table 5:** Mortality by precipitating factors GI: gastrointestinal

Precipitating factor	Patients with factor, n (%)	Deaths, n (%)
Infection	97 (48.2%)	37 (38.1%)
GI bleeding	46 (22.9%)	15 (32.6%)
Electrolyte imbalance	39 (19.4%)	11 (28.2%)
Constipation	33 (16.4%)	6 (18.2%)
Excess protein intake	21 (10.4%)	3 (14.3%)
Sedative use	18 (9.0%)	5 (27.8%)
No identifiable cause	27 (13.4%)	7 (25.9%)

Non-survivors had a higher mean total bilirubin level (5.1 ± 3.1 mg/dL) compared to survivors (3.6 ± 2.4 mg/dL, p = 0.01) and lower serum albumin levels (2.5 ± 0.5 g/dL vs. 2.9 ± 0.6 g/dL, p = 0.02). Coagulation was more deranged in non-survivors, with a higher INR of 2.2 ± 0.6 versus 1.8 ± 0.4 in survivors (p = 0.003). Renal function was significantly impaired in non-survivors, with mean creatinine levels of 1.8 ± 0.5 mg/dL compared to 1.2 ± 0.3 mg/dL in survivors (p < 0.001). Markers of systemic inflammation were also elevated in non-survivors, as evidenced by a higher white blood cell count (11,700 ± 3,200/mm³ vs. 9,200 ± 2,500/mm³, p = 0.005) and CRP levels (34.7 ± 15.6 mg/L vs. 23.5 ± 11.2 mg/L, p = 0.001). Arterial blood gas analysis showed significantly lower pH values in non-survivors (7.33 ± 0.07) compared to survivors (7.39 ± 0.04) (p < 0.001), consistent with a greater degree of acid-base disturbance in the non-survivor group (Table 6).

**Table 6:** Biochemical parameters by outcome INR: international normalised ratio; CRP: C-reactive protein; SD: standard deviation Independent t-test was used to compare the groups. A p-value <0.05 was considered statistically significant.

Parameter	Survivors (Mean ± SD)	Non-survivors (Mean ± SD)	p-value
Total bilirubin (mg/dL)	3.6 ± 2.4	5.1 ± 3.1	0.01
Albumin (g/dL)	2.9 ± 0.6	2.5 ± 0.5	0.02
INR	1.8 ± 0.4	2.2 ± 0.6	0.003
Creatinine (mg/dL)	1.2 ± 0.3	1.8 ± 0.5	<0.001
White blood cell count (/mm³)	9,200 ± 2,500	11,700 ± 3,200	0.005
CRP (mg/L)	23.5 ± 11.2	34.7 ± 15.6	0.001
pH (arterial)	7.39 ± 0.04	7.33 ± 0.07	<0.001

## Discussion

This retrospective study of 201 patients admitted with HE provides valuable insights into the clinical and biochemical predictors of in-hospital mortality. The findings we present underscore the significance of HE. The hospital mortality rate for our study was 28.4%, which is consistent with other research (indicating a 25%-35% mortality rate amongst patients with overt HE) [14]. People with high-grade encephalopathy (West Haven grades III or IV) were at a much higher risk. This finding accentuates the acute need to realize and handle patients with the manifestations of increasing mental deterioration [14]. It was found that the independent predictors identified here stated that at least the MELD score of 28 was the strongest predictor of mortality. This finding agrees with previous studies that related high MELD scores to a high risk of hepatic decompensation and multi-organ failure [15].

In non-survivors, the mean serum ammonia concentration (136.2 µmol/L) was significantly higher compared with survivors (103.1 µmol/L), and values of ≥120 µmol/L significantly predicted increased risk of death. While hyperammonemia is known to be a cause of HE, there remains discussion of its role as a predictor of mortality. We obtained results that show that increased concentration of ammonia does more than indicate the severity of HE; it also directly affects the adverse outcomes, probably through mechanisms like swelling of astrocytes, oxidative stress, or cerebral edema [16]. The presence of renal dysfunction based on creatinine thresholds, dialysis requirements, or acute deterioration in CKD was independently related to higher mortality, reinforcing the complex association between liver and kidney health in cirrhotic patients [17]. Hepatorenal syndrome and sepsis-associated acute kidney injury can appreciably increase it since both ammonia accumulation and proliferation of systemic inflammation due to a worsening of the renal function can occur in the course of these diseases [17]. A clear risk factor for poor patient outcomes was significantly lowered serum sodium (<130 mEq/L) [18]. Both microscopic and macroscopic studies have shown that serum sodium levels dropping below normal are connected to the violation of vasopressin regulation in advanced cirrhosis, causing cerebral edema and exacerbating HE symptoms [19]. Such observation is consistent with the accumulating evidence that disruptions in sodium balance have a decisive role in HE progression as well as mortality.

Our analysis of precipitating causes showed that infection, gastrointestinal bleeding, and disturbances of electrolytes represented the main risks of HE in our cohort [20]. Hyponatremia, observed in over 80% of those with electrolyte disturbances, is a well-known contributor to worsening HE due to its effect on cerebral edema and astrocyte swelling. Similarly, hypokalemia and hypocalcemia, though less frequent, can exacerbate HE by increasing ammonia production and impairing neuromuscular transmission. The high frequency of these imbalances underscores the need for vigilant electrolyte monitoring and correction in HE patients. Infection's dominance as a precipitant (48.2% of cases) and its associated 38.1% mortality, the highest among precipitants, underscores its role as a catalyst in the "perfect storm" of HE: systemic inflammation amplifies cytokine-mediated blood-brain barrier disruption while impairing ammonia detoxification [20]. This dual-hit mechanism explains why infection-associated HE had 1.8-fold higher mortality than constipation-related cases (18.2%). Crucially, this identifies a modifiable target; early antibiotic administration and source control could disrupt this cascade.

Even though the research involved a decent patient pool and rigorous statistical evaluation, the following major limitations persist. The retrospective design inherently carries the risk of selection bias and limits control over data completeness and accuracy. Some patients had missing imaging or laboratory data, and potential confounders such as nutritional status, lactulose use, time to treatment initiation, microbiome composition, or genetic polymorphisms in ammonia metabolism were not uniformly recorded. As our study focused on in-hospital mortality as a binary endpoint, survival analysis was not performed due to the lack of long-term follow-up data, which remains a recognized limitation. Finally, although the MELD score was a statistically significant predictor, its area under the curve (AUC) of 0.78 indicates only moderate discriminative ability, underscoring the need for more comprehensive predictive models. Furthermore, while this was a single-center study with a moderate sample size, multicenter validation would strengthen the generalizability of our findings.

## Conclusions

It is concluded that HE remains a critical complication of liver disease, associated with a substantial risk of in-hospital mortality. In this retrospective analysis of 201 patients, several independent predictors of mortality were identified, including a MELD score of 28 or higher, serum ammonia levels of 120 µmol/L or above, renal dysfunction, high-grade HE (Grade III or IV), and serum sodium levels below 130 mEq/L. These variables serve as important clinical markers for early risk stratification and targeted intervention. High-grade encephalopathy and systemic complications such as renal failure and hyponatremia were especially impactful on patient outcomes, underscoring the need for comprehensive and integrated management strategies.
